# Immunization with recombinant *Salmonella* expressing SspH2-EscI protects mice against wild type *Salmonella* infection

**DOI:** 10.1186/s12917-018-1404-5

**Published:** 2018-03-09

**Authors:** Maozhi Hu, Weixin Zhao, Hongying Li, Jie Gu, Qiuxiang Yan, Xiaohui Zhou, Zhiming Pan, Guiyou Cui, Xinan Jiao

**Affiliations:** 1 Jiangsu Co-innovation Center for Prevention and Control of Important Animal Infectious Diseases and Zoonoses, Yangzhou University, Yangzhou, Jiangsu 225009 China; 2 Jiangsu Key Laboratory of Zoonosis, Yangzhou University, Yangzhou, Jiangsu 225009 China; 3 College of Tourism & Cuisine (College of Food Science and Engineering), Yangzhou University, Yangzhou, Jiangsu 225009 China; 4 Department of Pathobiology and Veterinary Science, University of Connecticut, Storrs, CT 06269-3089 USA

**Keywords:** *Salmonella*, Caspase-1, SspH2-EscI, Mice, Protection

## Abstract

**Background:**

Enhancing caspase-1 activation in macrophages is helpful for the clearance of intracellular bacteria in mice. Our previous studies have shown that EscI, an inner rod protein of type III system in *E. coli* can enhance caspase-1 activation. The purpose of this study was to further analyze the prospect of EscI in the vaccine design.

**Results:**

A recombinant *Salmonella* expressing SspH2-EscI fusion protein using the promotor of *Salmonella* effector SspH2, X4550(pYA3334-P-SspH2-EscI), was constructed. A control recombinant *Salmonella* expressing SspH2 only X4550(pYA3334-P-SspH2) was also constructed. In the early stage of in vitro infection of mouse peritoneal macrophages, X4550(pYA3334-P-SspH2-EscI) could significantly (*P* < 0.05) enhance intracellular caspase-1 activation and pyroptotic cell death of macrophages, when compared with X4550(pYA3334-P-SspH2). Except for the intracellular pH value, the levels of reactive oxygen species, intracellular concentration of calcium ions, nitric oxide and mitochondrial membrane potential in macrophages were not significantly different between the cells infected with X4550(pYA3334-P-SspH2-EscI) and those infected with X4550(pYA3334-P-SspH2). Besides, only lower inflammatory cytokines secretion was induced by X4550(pYA3334-P-SspH2-EscI) than X4550(pYA3334-P-SspH2). After intravenous immunization of mice (1 × 10^6^ cfu/mouse), the colonization of X4550(pYA3334-P-SspH2-EscI) in mice was significantly limited at one week post immunization (wpi), when compared with X4550(pYA3334-P-SspH2) (*P* < 0.05). The population of activated CD8^+^T lymphocytes in mouse spleens induced by X4550(pYA3334-P-SspH2-EscI) was lower than that induced by X4550(pYA3334-P-SspH2) at 2–3 wpi, and the ratio of CD4^+^T cells to CD8^+^T cells decreased. The blood coagulation assay indicated that no significant difference was found between X4550(pYA3334-P-SspH2-EscI) and uninfected control, while X4550(pYA3334-P-SspH2) could induce the quick coagulation. Notably, immunization of X4550(pYA3334-P-SspH2-EscI) could limit the colonization of challenged *Salmonella* strains in the early stage of infection and provide more effective protection.

**Conclusion:**

The activation of caspase-1 in macrophages by EscI can be used in the design of live attenuated *Salmonella* vaccine candidate.

## Background

*Salmonella* is a facultative intracellular bacterium and common cause of foodborne illness, causing decreased breeding potential and increased fatality in host. During the early stage of infection, *Salmonella* can regulate the host cells’ defense mechanisms to ensure the survival of the invading bacteria by selectively secreting effectors through its type III secretion system (T3SS) [1–3]. Carriers of the bacteria contribute greatly to the propagation of the disease. Thus, the resistance of bacterial intracellular survival using innate immunity is a good choice to defence against *Salmonella* early infection [4, 5].

Macrophages play a critical role in defense against infection, whereas they are also the predominant host cells in the process of *Salmonella* infection [4]. Therefore, targeting the interaction of *Salmonella* and macrophages will help us explore the mechanism of resisting bacterial colonization in host cells. In innate immunity, intracellular nucleotide binding domain leucine-rich repeat-containing receptor (NLR) can recognize microbial components and then activate inflammasome signaling in macrophages [6]. During this process, the intracellular pro-caspase-1 is cleaved into the activated caspase-1 and subsequently triggers macrophage pyroptotic death [7, 8]. This pathway is beneficial for the defense against the colonization of intracellular bacteria in vivo [9, 10].

Studies have shown that many proteins of *Salmonella* can activate intracellular NLRC4 (NLR family, CARD domain containing-4) inflammasome response, but *Salmonella* can escape the inflammasome response during the process of infection [10]. The *Salmonella* strain that can enhance activation of inflammasome would inhibit their intracellular survival [11, 12]. As reported, recombinant *Salmonella* expressing fusion protein between the N-terminus of *Salmonella* SspH2 (SspH2 can be recognized by T3SS2) and the C-terminus of *E. coli* EscI (EscI can activate the NLRC4 inflammasome) (SspH2-EscI) can enhance the activation of inflammasome and limit its colonization in mice [5]. However, the function of SspH2-EscI as vaccine candidate in defense against *Salmonella* early infection is unclear. In the present study, a recombinant *Salmonella* expressing fusion protein SspH2-EscI using the promotor of SspH2 was constructed. The recombinant strain was tested for its ability to induce immune responses and defense against *Salmonella* infection in mouse.

## Methods

### Animals, plasmids and bacteria

Six-week-old female C57BL/6 mice with the average weight of 17 g per mouse were obtained from the Comparative Medicine Center of Yangzhou University (Yangzhou, China). This study was carried out in accordance with the regulations established by the Chinese Ministry of Science and Technology. The animal experiment protocol was approved by the Committee on the Ethics of Animal Experiments of Yangzhou University (Permit Number: 2007–0005). All surgery was performed under anesthesia intraperitoneally injected with sodium pentobarbital, 40 mg per kilogram mouse weight, and all efforts were made to minimize suffering.

Plasmid pYA3334, recombinant plasmid pYA3334-SspH2-EscI, *E. coli* X6212, attenuated *S*. Typhimurium strains X3730 and X4550 were used for the construction of recombinant bacteria as previously described [5]. Recombinant X4550(pYA3334) was used as control and wild type *S*. Typhimurium strain D6 isolated from the pig carcass in Yangzhou slaughterhouse was used to challenge mice for the protection assay. Bacterial strains were grown in Luria broth (LB) medium.

### Construction of recombinant *Salmonella* X4550(pYA3334-P-SspH2-EscI)

The genomic DNA of bacteria D6 were extracted using the high pure polymerase chain reaction (PCR) template preparation kit (Takara, Dalian, China) according to the manufacturer’s instructions. The P-SspH2 sequence containing 5′-terminal sequence (1–453 bp) of the *sspH2* gene and its promotor sequence was amplified from the D6 strain using the following primers: SspH2-F5 (5’-CCATGGAGTTGCCTGATACGGATGAAAACC-3′, forward) and SspH2-R3 (5’-GTCGAC**ACCGCCACC**TGTCCCGGATGCCCCT-3′, reverse). The purified PCR products of P-SspH2 and the recombinant plasmid pYA3334-SspH2-EscI were mixed for the overlap PCR splicing using the following primers: SspH2-F5 (forward) and EscI-R2 (5’-GAACAGTCGACCTACTTATCGTCGTCATCCTTG-3′, reverse). In the primers used in this study, the underlined segments indicate the restriction sites and the bold segments indicate the linker for overlap PCR. All PCR products were subsequently identified via agarose gel electrophoresis.

The construction of the recombinant bacteria was performed as previously described [5]. The recombinant bacteria were designated as X4550(pYA3334-P-SspH2-EscI). The PCR product P-sspH2 amplified from the D6 strain using the primers SspH2-F5 (forward) and SspH2-R4 (5’-GTCGACCTACTTATCGTCGTCATCCTTGTAATC**ACCGCCACC**TGTCCCGGAT-3′, reverse) was cloned into the plasmid pYA3334. The recombinant plasmid was named as pYA3334-P-SspH2 and the corresponding recombinant bacteria was named as X4550(pYA3334-P-SspH2).

### In vitro infection of mouse peritoneal macrophages

The in vitro infection experiment was performed as previously described [5, 13, 14]. Briefly, Peritoneal cells were collected and seeded on 96-well plates for culturing. Three hours later. The density of adherent cells was adjusted to 20,000 cells per well with RPMI 1640 complete medium without antibiotics. The strains X4550(pYA3334-P-SspH2-EscI), X4550(pYA3334-P-SspH2) and X4550(pYA3334) were added at an multiplicity of infection (MOI) of 100, respectively. After incubation for 30 min, the penicillin and streptomycin were added and the cells were remained in culture for different hours. The uninfected cells were used as controls.

To identify the enhanced activation of caspase-1 in macrophages, the intracellular caspase-1 activation were determined using FLICA™ staining. Mouse inflammatory cytokines in supernatants were quantified using cytometric bead array system (CBA). Both were analyzed by flow cytometry as previously described [5].

The cell morphology was observed and the lactate dehydrogenase (LDH) release was measured as previously described [5, 15].

To analyze the inpact on cell function, cells were collected and stained with rhodamine 123 (Rh123), Fluo-3 AM, DCFH-DA, DAF-FM DA and BCECF AM (Beyotime Institute of Biotechnology, China) for the assay of mitochondrial membrane potential (MMP), intracellular concentration of Calcium ions ([Ca^2+^]_i_), reactive oxygen species (ROS), nitric oxide (NO) and intracellular pH value ([pH]_i_), respectively [4, 16, 17]. All protocols were performed according to the manufacturer’s instructions.

### In vivo infection of mice

Bacterial immunization of mice was performed as previously described [5]. Briefly, Six-week-old C57BL/6 mice were intravenously injected with freshly cultured X4550(pYA3334), X4550(pYA3334-P-SspH2) and X4550(pYA3334-P-SspH2-EscI), respectively. Each mouse was immunized with 1× 10^6^ cfu using 100 μl phosphate-buffered saline (PBS) as vehicle. The mice intravenously injected with equivalent PBS were used as controls.

At different weeks post immunization (wpi), the blood of mouse was collected for coagulation observation.

To analyze the T lymphocytes responses, the splenic T lymphocytes of mice at 2–3 wpi were separated as previously described [18]. One part of splenocytes were stained simultaneously with PE-labeled anti-CD4, FITC-labeled anti-CD69 and APC-labeled anti-CD8a (PharMingen) for cell activation assay. The other part were stained simultaneously with PE-labeled anti-CD4, FITC-labeled anti-CD3 and APC-labeled anti-CD8a (PharMingen) for T lymphocyte subsets assay. After washing, cells were analyzed by flow cytometry. Five mice were used in each treatment.

The virulence of wild type *Salmonella* strain D6 was measured by determining a 50% lethal dose (LD50) 10 days after intraperitoneally administration of live bacteria. To examine the protective efficacy of the above recombinant bacteria, the immunized mice were intraperitoneally challenged with *Salmonella* strain D6 at 1 wpi. The bacterial colonization in spleen and liver of mice challenged with 1× 10^5^ cfu/mouse were counted by coating on the LB agar plate for culturing. After challenged with 5× 10^6^ cfu/mouse, surviving mice were counted for 15 days and the daily clinical signs including anorexia, diarrhea, depression, and mortality were recorded. Nine mice for each treatment were analyzed.

### Statistical analysis

Within each experiment, three to four replicate experiments were conducted for each treatment and the average value was calculated for final statistical comparisons. All statistical analyses were performed by *t*-tests using SPSS software (Version 13.0 for Windows, Chicago, IL). A value of *P* ≤ 0.05 was considered to be statistically significant.

## Results

### EscI enhanced caspase-1 activation in macrophages

**Fig. 1:**
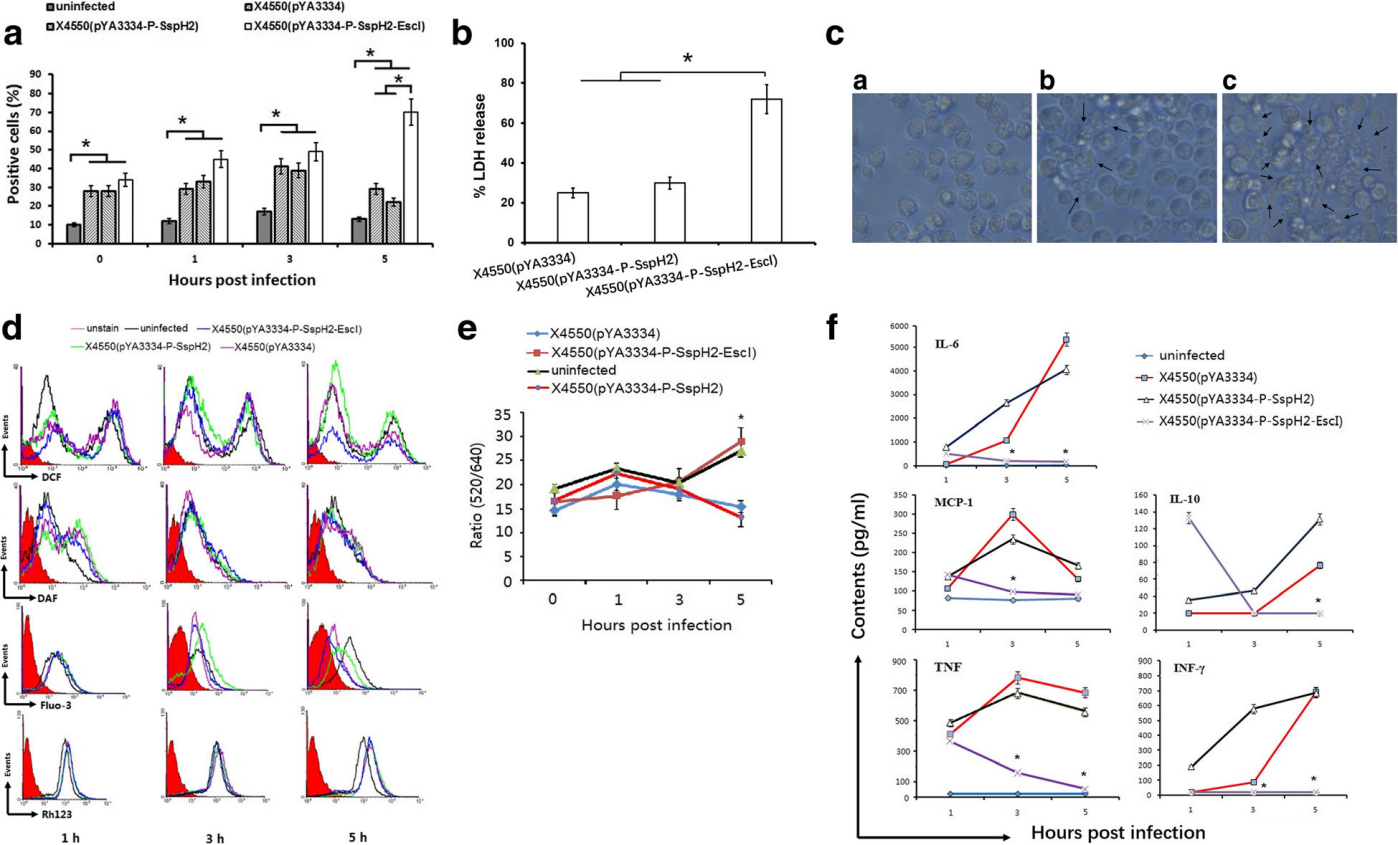
In vitro infection of mouse peritoneal macrophages. Six-week-old female C57BL/6 mouse peritoneal macrophages were seeded on 96-well plates for culturing. Three hours later, non-adherent cells were removed and cell density was adjusted to 20,000 cells per well with RPMI 1640 complete medium without antibiotics and the freshly cultured X4550(pYA3334), X4550(pYA3334-P-SspH2) or X4550(pYA3334-P-SspH2-EscI) were added with MOI 100. The cell plate was centrifuged to enhance the contact of bacteria with the cells and the infected cells were then incubated for 30 min. The supernatants containing uninfected bacteria were replaced with RPMI 1640 complete medium (100 μl/well) containing 100 U/ml penicillin and 100 μg/ml streptomycin prior to the start of the subsequent incubation. The uninfected cells were used as control. **a** Activation of intracellular caspase-1 at different hours post-infection (hpi) using FLICA staining; **b** Cytotoxicity assay by lactate dehydrogenase (LDH) release at 5 hpi; **c** Cell morphology observation at 5 hpi. a, uninfected control; b, X4550(pYA3334-P-SspH2) infection; c, X4550(pYA3334-P-SspH2-EscI) infection. **d** Cell function including reactive oxygen species (ROS), nitric oxide (NO), intracellular Ca^2+^ concentration ([Ca^2+^]_i_), and mitochondrial membrane potential (MMP) at 1, 3, 5 hpi stained with DCFH-DA, DAF-FM DA, Fluo-3 AM and rhodamine 123 (Rh123) respectively. **e** Intracellular pH value at 0, 1, 3, 5 hpi stained with BCECF-AM; **f** Supernatant cytokines levels at 1, 3, 5 hpi using cytometric bead array system kit. Results are representative of at least three independent experiments (**c**, **d**). *P < 0.05; X4550(pYA3334-P-SspH2-EscI) infection vs X4550(pYA3334-P-SspH2) or X4550(pYA3334) infection (**e**, **f**)

After in vitro infection of mouse peritoneal macrophages, flow cytometric assay indicated that all bacteria could induce the activation of intracellular caspase-1 at 1, 3, 5 h post infection (hpi), when compared with the uninfected control (*P* < 0.05). Notably, X4550(pYA3334-P-SspH2-EscI) induced significantly more caspase-1 activation at 5 hpi than X4550(pYA3334-P-SspH2) or X4550(pYA3334) (*P* < 0.05, Fig. 1a). LDH release assay showed that X4550(pYA3334-P-SspH2-EscI) induced higher cytotoxicity than X4550(pYA3334-P-SspH2) and X4550(pYA3334) at 5 hpi (*P* < 0.05, Fig. 1b). Cell morphological observation showed that more pyroptotic cells were found after infection with X4550(pYA3334-P-SspH2-EscI) at 5 hpi than infection with X4550(pYA3334-P-SspH2) or X4550(pYA3334) (Fig. 1c). No significant difference was found between X4550(pYA3334-P-SspH2) and X4550(pYA3334). These results suggested that the difference may be due to the expression of EscI, but not SspH2.

### EscI regulates [pH]_i_, but not ROS, [Ca^2+^]i, NO and MMP in macrophages

Cells were analyzed after being stained with different fluorescent dyes at 1, 3, 5 hpi. The levels of ROS, NO, [Ca^2+^]i and MMP are proportional to the intensity of intracellular DCF, DAF, Fluo-3 and Rh123. Flow cytometric assay showed similar changing trends of them among different infection groups, indicating that there were similar functional cellular responses of ROS, [Ca^2+^]i, NO and MMP in the early stage of infection (Fig. 1d). The ratio of fluorescence intensity (520 nm/640 nm) of BCECF-staining cells showed that no difference between X4550(pYA3334-P-SspH2-EscI) infection and uninfected control, while it was lower for X4550(pYA3334-P-SspH2) and X4550(pYA3334) infection than X4550(pYA3334-P-SspH2-EscI) infection at 5 hpi (Fig. 1e) indicating that X4550(pYA3334-P-SspH2) and X4550(pYA3334) regulate the [pH]_i_ of macrophages, but not X4550(pYA3334-P-SspH2-EscI). In this experiment, no significant difference was found between X4550(pYA3334-P-SspH2) and X4550(pYA3334).

### EscI inhibits inflammatory cytokines secretion in macrophages

Flow cytometric assay showed that the cytokines secretion induced by X4550(pYA3334-P-SspH2-EscI) were different from that induced by X4550(pYA3334-P-SspH2) or X4550(pYA3334) in the early stage of infection. At 1 hpi, all bacteria could induce the secretion of TNF, when compared with uninfected control. Notably, X4550(pYA3334-P-SspH2-EscI) induced higher level of IL-10 than X4550(pYA3334-P-SspH2) or X4550(pYA3334). With the time extension, the levels of IL-6, IL-10, IFN-γ, MCP-1 and TNF increased when infected with X4550(pYA3334-P-SspH2) or X4550(pYA3334), while that for X4550(pYA3334-P-SspH2-EscI) decreased (Fig. 1f). No significant difference was found between X4550(pYA3334-P-SspH2) and X4550(pYA3334) in this experiment.

### Immune responses induced by recombinant *Salmonella* expressing SspH2-EscI

At 1 wpi with 1 × 10^6^ cfu per mouse, the spleen of mice infected with X4550(pYA3334-P-SspH2) or X4550(pYA3334) showed swelling and dark color, when compared with uninfected control. No significant difference was found between X4550(pYA3334-P-SspH2-EscI) and uninfected control.

**Fig. 2:**
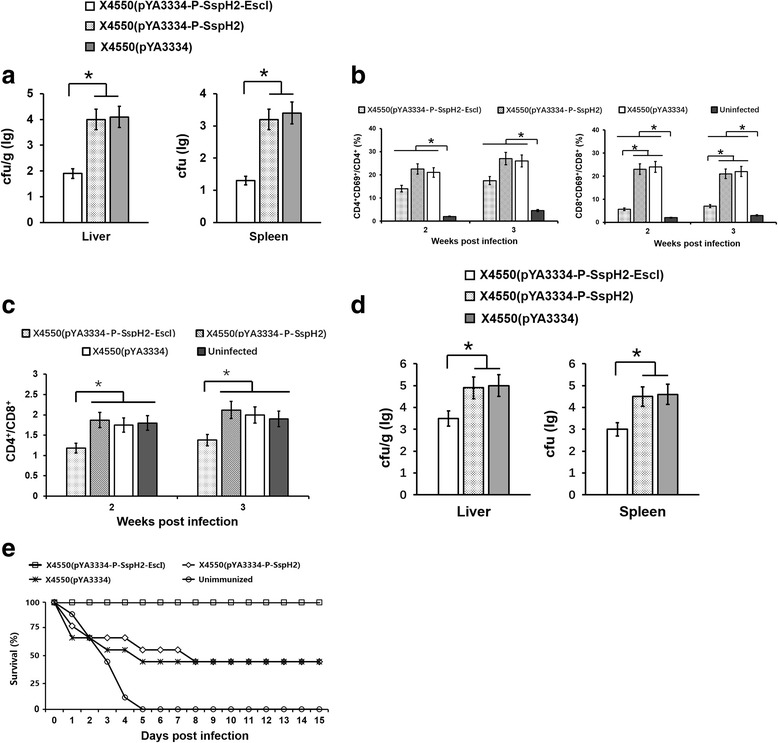
In vivo infection of mice. Six-week-old C57BL/6 mice were intravenously injected with either freshly collected X4550(pYA3334), X4550(pYA3334-P-SspH2) or X4550(pYA3334-P-SspH2-EscI), 1× 10^6^ cfu/mouse. The uninfected mice were used as control. Several weeks later, the bacterial counts (**a**) in mouse spleens and livers, the activation of splenic T lymphocyte subsets (**b**), the ratio of splenic CD4^+^/CD8^+^ T cells (**c**) were analyzed. Five mice were used in each treatment. One-week post immunization, the immunized mice were challenged intraperitoneally with wild-type *Salmonella* strain D6. The bacterial colonization (**d**) in mouse spleens and livers challenged with 1× 10^5^ cfu/mouse and the survival of mice challenged with 5 × 10^6^ cfu/mouse were counted (**e**) for immune protection assay. Nine mice were used for each treatment

Large amounts of bacteria were recovered from the spleen and liver of mice infected with X4550(pYA3334-P-SspH2) or X4550(pYA3334) at one day post infection (dpi), while only few was observed when infected with X4550(pYA3334-P-SspH2-EscI) (Fig. 2a). With the time extension, the colonization of X4550(pYA3334-P-SspH2) or X4550(pYA3334) increased, while colonization of X4550(pYA3334-P-SspH2-EscI) decreased. No X4550(pYA3334-P-SspH2-EscI) was observed in the spleen at 6 dpi.

At 2 and 3 wpi, flow cytometry was used to analyze the activation and differentiation of T lymphocytes in mouse spleens. The results showed that all bacteria could induce the activation of CD4^+^ and CD8^+^ T lymphocytes (*P* < 0.05), when compared with uninfected control. Notably, the population of activated CD8^+^T cells infected with X4550(pYA3334-P-SspH2-EscI) was significantly lower than that infected with X4550(pYA3334-P-SspH2) or X4550(pYA3334) (P < 0.05, Fig. 2b). The ratio of CD4^+^T cells to CD8^+^T cells decreased for X4550(pYA3334-P-SspH2-EscI) infection (*P* < 0.05, Fig. 2c), when compared with uninfected control.

At 1–3 wpi, the blood of mouse was collected for coagulation assay. X4550(pYA3334-P-SspH2) and X4550(pYA3334) could induce the quick coagulation, when compared with uninfected control. No significant difference of coagulation was found between that for X4550(pYA3334-P-SspH2-EscI) and uninfected control.

### Protective efficacy of recombinant *Salmonella* expressing SspH2-EscI

LD50 of *Salmonella* strain D6 was about 1 × 10^5^ cfu per mouse after intraperitoneal infection. One week after intravenous immunization (1 × 10^6^ cfu/mouse) with the above recombinant bacteria, the D6 strain was intraperitoneally injected to challenge the mice (1 × 10^5^ cfu/mouse). No significant clinical symptom was found for all mice. The amount of bacteria in the spleen and liver of mouse immunized with X4550(pYA3334-P-SspH2) or X4550(pYA3334) was about 14-fold and 35-fold greater than that immunized with X4550(pYA3334-P-SspH2-EscI) at 3 dpi, respectively (Fig. 2d). These result indicated that the immunization of X4550(pYA3334-P-SspH2-EscI) can limit the colonization of D6 strain.

When challenged with the dose of 5 × 10^6^ cfu per mouse, the X4550(pYA3334-P-SspH2-EscI)- immunized mice all survive, though some showed anorexia, diarrhea or depression. While about half of mice died for X4550(pYA3334-P-SspH2) or X4550(pYA3334) immunization (Fig. 2e). This indicated that X4550(pYA3334-P-SspH2-EscI) immunization can provide more effective protection against *Salmonella* challenge in the early stage of infection than X4550(pYA3334-P-SspH2) and X4550(pYA3334).

## Discussion

In 2002, Jürg Tschopp proposed the inflammasome complex as a molecular platform for caspase-1 activation [19]. The activation of caspase-1 can then induce pyroptotic cell death of macrophages [20]. Reports have shown that pyroptosis is a defense mechanism to clear intracellular bacteria [12, 21, 22]. Thus, the mechanism of inflammasome responses could be potentially used in the design of live vaccine candidates [12, 23]. In the past decade, a handful of inflammasomes that detect specific microbial challenges have been reported [20]. For example, in the early stage of *Salmonella* infection, NLRC4 inflammasome can be activated by *Salmonella* proteins PrgJ or flagellin [15, 24, 25]. However, *Salmonella* can escape the NLRC4 detection in the late stage of infection for its survival in macrophages. It is suggested that the *Salmonella* strain with the ability to enhance caspase-1 activation can strengthen the cell’s defense against *Salmonella* infection [23]. Our previous studies have shown that the recombinant *Salmonella* expressing fusion protein SspH2-EscI under the plasmid promoter *P*_*trc*_ can enhance NLRC4 inflammasome signaling and be completely cleared in vivo [5]. This will be beneficial for the live *Salmonella* vaccine design based on the inflammasome mechanism.

*Salmonella* strain X4550 is attenuated and usually used as a vector to transport exogenous antigen to promote immunity [5]. In this study, X4550(pYA3334-P-SspH2) and X4550(pYA3334) was selected as control to analyze the activation of caspase-1 in macrophages. In order to survive in the host cells, *Salmonella* can selectively secret cytoplasmic effectors through its type III secretion system (T3SS) to regulate cell function [2]. Accordingly, the expression of effector proteins may be regulated by the promoter of itself [26]. Thus, in this experiment, the SspH2 promoter *P*_*sspH2*_ was inserted into the 5′-terminus of *sspH2-escI* for the construction of recombinant plasmid pYA3334-P-SspH2-EscI and the recombinant *Salmonella* strain X4550(pYA3334-P-SspH2-EscI) was used to infect macrophages. The assay of caspase-1 activation, LDH release and cell morphological observation indicated that *P*_*sspH2*_ can initiate the expression of SspH2-EscI, which enhance the activation of caspase-1 in macrophages and then induce the pyroptotic cell death of macrophages. After intravenous infection of mice, the colonization of X4550(pYA3334-P-SspH2-EscI) in mouse spleens and livers was significantly decreased and no bacteria was found one-week later, in contrast colonization of X4550(pYA3334-P-SspH2) and X4550(pYA3334) was increased. Besides, the recombinant bacteria X4550(pYA3334-P-SspH2) and X4550(pYA3334) could induce low level of caspase-1 activation and no significant difference was found between them in all experiments of this study. These indicated that the enhanced activation of caspase-1 in macrophages is due to the expression of EscI.

Pyroptosis is a form of programmed necrosis which is different from apoptosis. It is lytic, featuring cell swelling and large bubbles blowing from the plasma membrane. In the process of pyroptosis, the pathogen replication niche is disrupted and the intracellular bacteria is directly killed through pore-induced intracellular traps [20, 22, 25, 27]. The pyroptotic cell death process may be accompanied with some changes of physiological function in host cells [28–30]. However, the impacts of pyroptosis on cell function, such as MMP, [Ca^2+^]i, ROS, NO and [pH]i, are rarely reported. In this experiment, we found that the changing trends of MMP, [Ca^2+^]i, ROS and NO in host cells induced by X4550(pYA3334-P-SspH2-EscI) were similar to that induced by X4550(pYA3334-P-SspH2) and X4550(pYA3334). This indicated that the pyroptotic cell death process does not change the MMP, [Ca^2+^]i, ROS and NO in host cells. The [pH]i level of host cells usually decrease to defense against bacteria in the process of *Salmonella* infection [4]. In this experiment, X4550(pYA3334-P-SspH2) and X4550(pYA3334) induced lower level of [pH]i, when compared with uninfected control. No significant difference was found between X4550(pYA3334-P-SspH2-EscI) and uninfected control. This may be due to EscI-activated pyroptosis, which limited the colonization of bacteria in host cells.

The secretion of inflammatory cytokines in the early stage of infection are beneficial for the defense against bacteria. In the process of *Salmonella* infection, on one hand, the host cells will secret inflammatory cytokines to control infection, on the other hand, *Salmonella* has evolved to survive in the inflammatory microenvironment [4]. In this study, the low level of IL-6, IL-10, IFN-γ, MCP-1 and TNF secretion induced by X4550(pYA3334-P-SspH2-EscI) may be due to the clearance of bacteria induced by EscI-activated pyroptosis.

The clearance of bacteria induced by pyroptosis occurs in the early stage of infection and no effective adaptive immune responses are produced at this time [5]. Early clearance of bacteria may influence the production of subsequent immunity. It is shown that the *Listeria monocytogenes* strain engineered to activate the NLRC4 inflammasome is severely attenuated and cannot induce effective immunity [31]. It is less immunogenic for CD8^+^ T cell responses than wild type *L. monocytogenes* [32]. In this study, at 2 and 3 weeks post intravenous infection of mice, we also found that all bacteria could induce the activation of CD4^+^ and CD8^+^ T lymphocytes, but X4550(pYA3334-P-SspH2-EscI) induced significantly lower level of CD8^+^T cells activation than X4550(pYA3334-P-SspH2) or X4550(pYA3334). Besides, the ratio of CD4^+^ T cells to CD8^+^ T cells decreased for X4550(pYA3334-P-SspH2-EscI) infection. These results verified that the enhanced activation of inflammasome can decrease the production of adaptive immunity due to the clearance of bacteria in the early stage of infection. The detailed mechanism needs to be further explored in the future.

Systemic bacterial infections are often associated with hemostatic changes that disrupt the coagulant/anticoagulant balance [33]. We showed that X4550(pYA3334-P-SspH2) and X4550(pYA3334) could induce the quick coagulation, while no difference was found between that for X4550(pYA3334-P-SspH2-EscI) and uninfected control. This may be associated with the quickly clearance of bacteria in mice.

Due to the clearance of bacteria, the inflammasome pathway has been hypothesized to be used in the design of live attenuated vaccine [11, 12]. NLRC4 can be activated by flagellin and *L. monocytogenes* can evade NLRC4 by repressing flagellin expression. The recombinant *L. monocytogenes* strain with forced expression of flagellin in the host cell cytosol can hyperactivate caspase-1 and is preferentially cleared via NLRC4 detection. The recombinant strain can confer protective immunity in mice against lethal challenge with *L. monocytogenes* [12]. We similarly showed that the recombinant *Salmonella* expressing SspH2-EscI could enhance the activation of caspase-1 and pyroptosis. One week post intravenous inmmunization, we found more effective protective immunity against lethal challenge with *Salmonella* when immunized with X4550(pYA3334-P-SspH2-EscI) than X4550(pYA3334) or X4550(pYA3334-P-SspH2). This indicated that the inflammasone pathway can be used in the design of live attenuated *Salmonella* vaccine, though the adaptive immune responses is decreased.

In this study, intravenous infection pathway was used to analyze the function of caspase-1 activation enhanced by EscI on the protection of mice against *Salmonella* infection. The inflammasome signaling is mainly studied in macrophages. *Salmonella* can colonize in different cells, such as B cells, T cells, neutrophilic granulocytes, monocytes and dendritic cells [34]. Different immune pathway may induce different responses in the early stage of infection due to the different cell types. Thus, whether the other immune pathways, such as oral or intraperitoneal injection, also produce the similar protection against *Salmonella* infection need to be further studied.

## Conclusions

A recombinant *Salmonella* expressing SspH2-EscI fusion protein using the promotor of SspH2 could enhance the activation of caspase-1 in macrophages and protect mice against *Salmonella* challenge. This indicated that the inflammasone pathway can be used in the design of live attenuated *Salmonella* vaccine.

## Abbreviations

[Ca^2+^]_i_: intracellular concentration of Calcium ions
[pH]_i_: intracellular pH value
CBA: Cytometric bead array system
dpi: day post infection
hpi: hour post infection
LB: Luria broth
LD50: 50% lethal dose
LDH: Lactate dehydrogenase
MMP: mitochondrial membrane potential
MOI: multiplicity of infection
NLR: Nucleotide binding domain leucine-rich repeat-containing receptor
NLRC4: NLR family, CARD domain containing-4
NO: nitric oxide
PBS: Phosphate buffer saline
PCR: Polymerase chain reaction
Rh123: rhodamine 123
ROS: reactive oxygen species
T3SS: Type III secretion system
wpi: week post immunization
